# Leveraging patient experience data to guide medicines development, regulation, access decisions and clinical care in the EU

**DOI:** 10.3389/fmed.2024.1408636

**Published:** 2024-05-23

**Authors:** Diogo Almeida, Denise Umuhire, Rosa Gonzalez-Quevedo, Ana António, Juan Garcia Burgos, Patrice Verpillat, Nathalie Bere, Bruno Sepodes, Carla Torre

**Affiliations:** ^1^Laboratory of Systems Integration Pharmacology, Clinical and Regulatory Science, Research Institute for Medicines (iMed.ULisboa), Lisbon, Portugal; ^2^Faculdade de Farmácia, Universidade de Lisboa, Lisbon, Portugal; ^3^Data Analytics and Methods Task Force, European Medicines Agency, Amsterdam, Netherlands; ^4^Public and Stakeholders Engagement Department, European Medicines Agency, Amsterdam, Netherlands; ^5^Referrals Office, Quality and Safety of Medicines Department, European Medicines Agency, Amsterdam, Netherlands; ^6^Regulatory Practice and Analysis, Medsafe—New Zealand Medicines and Medical Devices Safety Authority, Wellington, New Zealand

**Keywords:** patient experience data, patient-reported outcomes, patient preferences, regulatory, decision-making, challenges, opportunities, guidance

## Abstract

Patient experience data (PED), provided by patients/their carers without interpretation by clinicians, directly capture what matters more to patients on their medical condition, treatment and impact of healthcare. PED can be collected through different methodologies and these need to be robust and validated for its intended use. Medicine regulators are increasingly encouraging stakeholders to generate, collect and submit PED to support both scientific advice in development programs and regulatory decisions on the approval and use of these medicines. This article reviews the existing definitions and types of PED and demonstrate the potential for use in different settings of medicines’ life cycle, focusing on Patient-Reported Outcomes (PRO) and Patient Preferences (PP). Furthermore, it addresses some challenges and opportunities, alluding to important regulatory guidance that has been published, methodological aspects and digitalization, highlighting the lack of guidance as a key hurdle to achieve more systematic inclusion of PED in regulatory submissions. In addition, the article discusses opportunities at European and global level that could be implemented to leverage PED use. New digital tools that allow patients to collect PED in real time could also contribute to these advances, but it is equally important not to overlook the challenges they entail. The numerous and relevant initiatives being developed by various stakeholders in this field, including regulators, show their confidence in PED’s value and create an ideal moment to address challenges and consolidate PED use across medicines’ life cycle.

## Introduction

Recent years have seen an increased focus on patient engagement and on data collected directly from patients, with the objective of implementing patient-centric decision-making across the medicines’ life cycle. This paradigm-shift from disease-centered to patient-centered originates from the notion that patients are the experts in their own experience regarding their conditions and treatments (1). Moreover, since patients are the end users of medicines, it is fair to assume that patients should participate more in the decisions that directly affect their medical care. One way of supporting this principle is by integrating Patient Experience Data (PED) into medicines development early on, so these data can be analyzed and submitted to regulators for the scientific evaluation on benefit–risk leading to approval of a medicine. Analysis of these data can also generate evidence regarding patients’ experiences, preferences, needs and reported outcomes that could not only guide more downstream processes leading to medicines reimbursement and access decisions by healthcare systems, but also optimize clinical care (2).

Various stakeholders, including regulators, have been concentrating their efforts into examining obstacles to the integration of PED in medicines development and also regulatory decision-making (3). As an example, the European Medicines Agency (EMA) organized a workshop in 2022 (4) to identify ways to improve evidence generation through PED, which would support a key priority for the EU Medicines Regulatory Network to increase patient access to medicines. A consensus on what is considered PED in the EU was reached with the collaboration of regulators, patients, healthcare professionals, industry representatives and academics, and a tentative EU definition of PED was proposed as “*data collected* via *a* var*iety of patient engagement activities and methodologies to collect patients’ experience of their health status, symptoms, disease course, treatment preferences, quality of life and impact of health care*” (4).

In this context, it is also crucial to clarify the definition of Patient Engagement (PE) and how important it is for exploiting the collection and use of PED. The 2022 EMA workshop defined PE as “*all activities involving interaction with patients to gather their experience on disease, preferences, outcomes and treatments*” (4). Accordingly, augmented patient engagement through validated methodologies could, in turn, increase the quantity, quality and use of PED in regulatory processes, such as scientific advice on companies’ proposed plans to develop new medicines and further decisions on benefit–risk assessment of medicines (2, 4). Indeed, the scientific advice provided by EMA to medicine developers on their study design plans at early stages in medicines development is an example of the added value of capturing patients views in regulatory processes, and how these can help shape and have concrete impact on medicines’ development plans. A fifth of the scientific advices with regulatory recommendations provided by EMA to companies between 2017 and 2020 were shaped following patient input. Additionally, concerning the cases where no modifications based on patient input were made, patients agreed with the Agency’s advice on the proposed development plans in 90% of these cases (5). Thus, patients’ experience might add value to early advice regulatory processes, and offers an opportunity for more systematic use of the different types of PED in regulatory decision-making, underscoring the need for further promotion.

Therefore, this review aims to map the existing PED definitions, data collection methodologies and application of different types of PED, namely Patient-Reported Outcomes (PRO) and Patient Preferences (PP) and illustrate how these data can be used in the different phases of medicines’ life cycle. Additionally, and considering the recent advances in the field, some challenges and opportunities regarding the implementation of PED in regulatory decision-making will be discussed, focusing on aspects of guidance, methodological limitations and digitalization.

## Types of patient experience data and collection methods

### Patient engagement

Patient engagement in the development and regulation of medicines is an increasingly important element that aims to incorporate the views of patients throughout medicines’ life cycle, from research and development to regulatory approval and post-marketing surveillance. This approach recognizes patients as key actors whose insights and perspectives can significantly contribute to the safety, efficacy and acceptability of medicines (4–6). Although patient engagement is a relatively new field, experience to date allows us to describe some of the ways in which patient engagement is being integrated into the development and regulation of medicines (4).

By participating in the design of clinical trials, patients can provide valuable input at the design stage, helping researchers to prioritize outcomes that are most relevant to patients, select patient-friendly study protocols and improve recruitment strategies. Patients can also help develop informed consent materials to ensure that the information provided to participants is clear, understandable and responsive to patient concerns and preferences (7).

During the drug development process, patient advocates can work with pharmaceutical companies, regulatory agencies and even with the academic sector to provide insights into the lived experience of their condition, including treatment preferences, unmet needs and tolerability of potential side effects. This input can inform drug development strategies and decision-making processes (2, 5). In particular, regulatory agencies are increasingly involving patients in scientific advisory groups, public hearings and consultations to gather patient perspectives on benefit–risk assessments, prescribing requirements and post-marketing surveillance plans. Patient engagement continues after regulatory approval through initiatives such as patient registries, adverse event reporting systems and other measures that enable monitoring of treatment effectiveness and safety in real-world settings (2, 5).

Health technology assessment (HTA) agencies are also increasingly involving patients in their processes, taking into account their experiences with medical interventions, their views on health-related quality of life measures, and value assessments that inform reimbursement decisions (2). Particularly, the introduction of the Joint Clinical Assessment procedure in Europe, a centralized European framework for assessing clinical evidence within HTA assessments, is expected to promote PE in the HTA evaluation. In this line, EUnetHTA issued a guidance about patient engagement in 2023 (“Guidance on Patient and Healthcare Professional Involvement”) establishing a framework for the involvement of external experts, in which patients are included (8). The EU Health Technology Assessment regulation, including Joint Clinical Assessment, represents the most significant change in the European healthcare landscape in recent years. However critically, pricing and reimbursement decision-making remains the responsibility of individual member states.

To improve the incorporation of patient engagement in decision-making, a collaborative, multistakeholder approach is essential. An example of such collaborative efforts is the FDA/EMA Patient Engagement Cluster, established in 2016 to facilitate regular exchange of best practices regarding patient engagement throughout the medicine’s life cycle (9).

In 2018, the European Patients’ Academy on Therapeutic Innovation (EUPATI) issued a set of guidance documents to support the implementation of patient engagement among key stakeholders. Firstly, the organization clarifies how the term “patient” can be use more precisely to reflect different inputs, by establishing definitions for “individual patients,” “carers,” “patient advocates,” “patient organization representatives” and “patient experts.” Additionally, for regulators and regulatory agencies, the EUPATI suggests patient participation through initiatives such as: a network of patient organizations; a forum established within the regulatory authority composed by patient organizations; the creation of a group of individual patients expert in their disease and its treatment; cooperation in the field of communication for information dissemination; a dedicated program for capacity-building, namely about the regulatory system, and financial support for patients contributing to the regulator’s activities (10).

By integrating patient engagement into the development and regulation of medicines, stakeholders can ensure that medical products are developed, evaluated and regulated in a way that reflects patients’ needs, preferences and values. This patient-centered approach promotes transparency, accountability and trust in the healthcare system, ultimately resulting in improved health outcomes and quality of life for patients (4, 6, 10).

### Patient-reported outcomes

According to the EMA, Patient Reported Outcomes (PROs) “refer to a health/treatment outcome reported directly by the patient without the interpretation of a clinician or another person” (4). This implies that the patient’s experience is captured without modification or interpretation by a healthcare professional or anyone else (11, 12).

PRO data are collected through patient-reported outcome measures (PROMs). PROMs are instruments (e.g., questionnaires or diaries) filled out by patients themselves as a self-report and have to be validated (12, 13). Therefore, PROMs may provide helpful information regarding subjective outcomes/concepts such as symptoms (e.g., pain, fatigue, or nausea), mental functioning, physical functioning, well-being, adherence to treatment, satisfaction with treatment, and treatment preferences (11–13).

PROMs can be classified as generic or disease-specific. Generic PROMs can be used irrespectively of disease or patient, and they aim to measure single or multidimensional health concepts such as cognitive function, performance status, symptoms (including pain). Additionally, generic PROMs can also be applied in healthy subjects, for example, to evaluate overall health, a nontreatment intervention study, or a method to assess a PROM. Since they can apply to various patient populations, PROMs enable the comparison across different treatments or groups of patients under different environments/contexts and conditions. Nevertheless, generic PROMs lack sensitivity, can be less responsive to change and may fail to capture relevant disease-specific aspects when compared to disease-specific PROMs (12–14).

On the other hand, disease-specific PROMs provide a quantification of the impact of a specific disease. These instruments tend to be of higher relevance and responsiveness to changes, providing more comprehensive information on specific aspects of a disease. Nevertheless, they do not allow comparisons with general population data, across different patient populations and distinct diseases or conditions (11, 12, 14–17). Thus, it is fundamental to select PROMs (generic or disease-specific) that are fit-for-purpose considering the study’s objectives and the characteristics of the population subject to analysis (12). Table 1 summarizes the main advantages, limitations as well as gives examples of both generic and disease-specific PROMs.

**Table 1:** Advantages, limitations and examples of generic and disease-specific Patient-Reported Outcome Measures.

	Generic PROMs	Disease-specific PROMs
Advantages	Allow for comparison across distinct treatments or groups of patients.	Tailored to a specific disease/condition.
	Allow for comparison with the general population data that can be used to interpret scores.	May have higher relevance and responsiveness to change.
		May be more sensitive to particular domains of a disease.
Limitations	May include less relevant items, or on the contrary, exclude relevant items.	Do not allow for comparisons across patient populations with different diseases or conditions.
	May be less sensitive to changes within the specific domains to the disease.	May fail to identify relevant general domains and unexpected treatment-related toxicities.
		Do not allow for comparison with the general population data.
Examples (12, 13)	EuroQoL-5 (EQ-5D); 36-Item Short Form Survey (SF-36); Nottingham Health Profile (NHP); and Sickness Impact Profile (SIP); Patient-Reported Outcomes Measurement Information System (PROMIS); World Health Organization Quality of Life-100 (WHOQOL-100); Visual Analog Scale (VAS); 12-Item Short Form Survey (SF-12).	EORTC QLQ-C30 (European Organization for Research and Treatment); Functional Assessment of Cancer Therapy-General (FACT-G); International Index of Erectile Function (IIEF); National Eye Institute Visual Functioning Questionnaire; Pediatric Asthma Quality of Life Questionnaire (PAQLQ), Quality of Life in Epilepsy (QOLIE); Rotterdam Symptom Checklist (RSCL); Lung Cancer Symptom Scale (LCSS); MD Anderson Symptom Inventory (MDASI); Functional Assessment of Cancer Therapy Hepatobiliary Cancer Symptom Index—8 Item Version (FHSI-8); Functional Assessment of Cancer Therapy—Ovarian Symptom Index (FOSI); Functional Assessment of Chronic Illness Therapy (FACIT); Patient-Reported Outcomes version of the Common Terminology Criteria for Adverse Events (PRO-CTCAE).

Considering both advantages and limitations of each category of PROMs, some authors recommend the combined administration of generic and disease-specific PROMs for a more thorough appraisal of the outcomes to be studied (14). Hybrid measurement systems, also known as modular packages, use a generic health measure plus complementary disease-specific instruments. For example, the FACIT (Functional Assessment of Chronic Illness Therapy) system comprises a generic Health-Related Quality of Life (HRQL) measure and complementary disease-specific subscales. Most importantly, this modular approach may also improve comparability of PRO data (14, 15, 18).

At a data collection level, PROMs can be a questionnaire, a self-report, or an interview, among other strategies; in either case, only the patient’s answers are considered, unless this is not possible, in which case caregivers may report these outcomes (12). The development of a PROM should start with the construction of a conceptual framework in which simpler concepts can be properly clustered into more complex groups for measurement purposes (11, 19). This conceptual framework for the development of a PROM should consider, at its core, the desired claim, and it should not only be developed based on literature review, experts’ and physicians’ knowledge, but also stem from patient input and experience.

During this development process, patient input is vital to adapt and confirm the conceptual framework and can be obtained through a variety of methods, such as exploratory patient interviews, focus groups and other feedback strategies (19–21). Patient advocates and experts can attest if the concept is meaningful; suggest other domains to be included in the instrument; provide feedback to develop an instrument measure that consider diminishing missing data strategies; share cultural and linguistic details to be considered; advise on the medium and technologies used in the data collection phase; and even participate in the dissemination of the PROM, promoting its use among their networks (19). Thus, involving more differentiated patient input might be of added value in this development phase of a PROM (19, 21). Overall, the PROM development framework will provide the rationale regarding what outcomes to measure and how this is to be accomplished. It should also determine the target population and research application, which will, in turn, define the instrument’s characteristics (20, 22, 23).

Measurement property testing is also required, which will confirm, prior to the creation of the instrument itself, that the findings drawn from the application of the PROM are valid and relevant (19, 21). Content, criterion and construct validity correspond to three measurement properties that are often assessed. Table 2 summarizes the definitions of these measurement properties and presents some examples of their application (24).

**Table 2:** Examples of the practical application of content, criterion and construct validity.

Measurement property	Definition	Example
Content validity	The degree to which the instrument accurately assesses the intended concept it was designed to measure (24).	If a questionnaire is developed as a PROM to evaluate how one therapeutic causes breathlessness, the content validity of this instrument would be the extent to which it would measure breathlessness, instead of anxiety or shallow breathing, or any other situations that could be mistaken with the actual claim it is intended to measure (22, 23).
Criterion validity	The extent to which a score of a particular instrument relates to a gold standard (24).	To determine a threshold in the PROM 9-item Patient Health Questionnaire (PHQ-9), scores were compared against the gold standard for diagnosing major depression, which involves an interview conducted by a healthcare professional following the Diagnostic and Statistical Manual of Mental Disorders (DSM) guidelines (25).
Construct validity	The extent to which a score given through one instrument matches consistently with the hypothesis proposed (24, 26).	In individuals with COPD, it is anticipated that those with lower treadmill exercise capacity will typically experience more dyspnea in daily life compared to those with higher exercise capacities (27).

Content validity corresponds to the degree to which the instrument accurately assesses the intended concept it was designed to measure (24). To prove its content validity, robust evidence should be gathered demonstrating that the instrument measures its purpose and that its items and domains are appropriate regarding the concept intended to be measured, population, and use (24). When it comes to a PROM, the qualitative work with patients is relevant since a PROM is meant to measure important concepts from the patient’s perspective. This way, the PROM should be built according to the patient’s comprehensiveness and perspective, and not according to clinicians and other stakeholders. Criterion validity, a related concept, must also be addressed as it intends to describe how the score of a particular instrument relates to a gold standard, demonstrating how similar the instrument used is to the gold standard instrument (24).

Construct validity corresponds to another type of measurement properties and is established as the extent to which a score given through one instrument matches consistently with the hypothesis proposed, regarding the claims being measured, and represents the relationship between the score and the theoretical claim (24, 26). A PROM must also show reliability, a measurement property which also contributes to its validity. Reliability means that the instrument will reproduce consistent results over time, if applied at different time points, for example throughout a clinical trial (24).

These instruments should also be able to detect if clinically important changes have occurred in an outcome, according to the study’s objectives. The degree to which a PROM can detect the changes of the measures over time should be well defined in advance (28, 29). Therefore, another relevant measurement property of the PROM is the minimal clinically importance difference (MCID), which can be defined as a measure of the smallest change in an outcome that patients perceive as important and may therefore require a change in patient’s management (30). To illustrate this concept, while MCID has been used to help healthcare professionals to assess if an intervention might cause a clinically important change, applying it to PROM allows patients to participate in the definition of what they find to be a meaningful change (30). The use of MCID is progressively expanding to comprehend the clinical effectiveness of a specific treatment, establish clinical practice guidelines, and accurately interpret trial outcomes. Despite this, different methods for MCID calculation (e.g., anchor-based methods, distribution-based methods) can result in heterogeneous results which can make the evaluation of the treatment’s effectiveness difficult. Consequently, research has been developed to calculate MCID thresholds for common PROMs to obtain more accurate results (30, 31).

After having reached the initial stages of PROM development, cognitive interviews are usually carried out in order to check how the items incorporated in the PROM are assessed by individual patients or carers in terms of clarity and relevance (32, 33). These cognitive interviews with patients then allow researchers to compare if the understanding of the instrument matches the idea that instigated its development and whether issues of literacy, jargon, technical language, or culture-specific constructs exist. The research team organizes these interviews until a saturation of information is reached, i.e., there is no additional information if more interviews were conducted. Consequently, the results and content obtained from the cognitive interviews is then considered during a revision phase where items can be altered accordingly (33). Patient organizations may play a crucial role in identifying individual patients or carers that consent to participate in these cognitive interviews.

Finally, PROM’s revision does not end in the development phase. During its use, additional clinical data, other patients’ inputs and considerations regarding changes of the environment can be gathered to instigate new revision processes, assuring the quality parameters that a PROM needs to reach. As an example, if a cognitive PROM’s item assesses the patients’ capacity to make a phone call, this indicator might need to be reviewed to start considering a scenario where patients use a smartphone, instead of a traditional landline phone, since both represent different cognitive abilities (33).

### Patient preferences

Patient Preferences (PP), in the medicines’ regulatory setting, can be summarized as instruments capable of indicating “*how desirable or acceptable is to patients a given alternative or choice among all the outcomes of a given medicine*” (4). PP information may complement traditionally collected safety and efficacy data, ensuring that the patients’ preferences, needs, and values will guide decision-making based on what patients are disposed to consent in terms of benefits and harms. Therefore, it is anticipated that patient-centered decisions will be more trusted by patients, clinicians, and the general public. Such decisions might also increase patient satisfaction, by meeting their needs and expectations, and ultimately improve their adherence to the treatment, leading to better health outcomes and effectiveness (34).

In this context, PP can generate different inputs when compared to PRO data. Notwithstanding, PP information is able to illustrate what outcomes are a priority and what medical needs are required to be met. Moreover, PP might also be able to promote and highlight the need of shared decision-making, resulting in outcomes more relevant to patients and optimized transparency (35). Concretely, a study by Fifer et al. in the context of multiple myeloma illustrates that patient preferences may vary from patient to patient, while professionals involved in patient care may place different value on patients’ preferred treatment outcomes (36).

PP information can be collected through mainly two different methods. Preference exploration methods (qualitative) collect in-depth descriptive data about patient experiences, perspectives, and the treatment attributes that are most important to them. Qualitative research is usually unstructured or semi-structured, such as individual interviews, focus groups and open-ended survey questions (37, 38). In turn, patient preference elicitation methods (quantitative) quantify patient preferences in a structured manner. They collect numerical data that assesses the relative weights assigned to different attributes and which compromises patients are willing to accept, allowing to statistically detect preference heterogeneity. Discrete choice experiments (DCE), threshold technique, swing-weighting, and best-worst scaling (BWS) are some examples of preference elicitation methods (39). Table 3 summarizes some examples of PP exploration and elicitation methods.

**Table 3:** Examples of PP collection methods.

Category	Nature	Description	Examples
Exploration (37, 38)	Qualitative	Collection of descriptive data about patients’ experiences and preferences.	(Semi-)structured interview
			In-depth individual interview
			Complaints procedures
Elicitation (39)	Quantitative	Structured and systematic quantification of patient preferences.	Discrete choice experiments (DCE)
			Swing-weighting
			Threshold technique
			Best-worst scaling (BWS)

In the context of an HTA study to establish the value of PP studies, a research program conducted by the National Institute for Health and Care Excellence (NICE) and the Myeloma UK patient charity identified DCE as the most used approach for eliciting patient preferences, due to its robustness and similarity to real-life decisions (40). This method is used in several studies to explain or predict a choice from a set of mutually exclusive alternatives (40–42). In the DCE approach, patients are asked to choose their preferred option, based on the attributes and their respective levels (effectiveness, duration of treatment benefit, risk of mild and/or serious side effects and mode of administration, for example). There may also be an “opt-out” option which allows the patient not to choose any alternative if neither are acceptable, providing a more accurate picture of the expected uptake of that treatment (37, 41–44). Therefore, this method is useful not only to assess the relative importance of treatment attributes, but also to measure the highest level of risk a patient would be willing to tolerate in return for a specific benefit (43).

Nevertheless, DCE is not universally applicable. For example, it is not a suitable method when there are many attributes to consider, when patients cannot process a large amount of information or require approaches of easier comprehension, or in case of small samples which do not allow valid statistical analysis, such as rare diseases (45, 46).

Although, PP collection methods can be categorized as either exploration or elicitation methods, they can also be classified as revealed-preference or stated-preference. Revealed-preference methods rely on observing real-world choices and behaviors to draw conclusions. Examples of revealed-preference methods encompass patient-preference trials and direct inquiries within clinical trials. Conversely, in stated-preference methods, patient preferences are elicited through hypothetical experiments. Stated-preference comprises methods such as direct assessment questions, DCE, threshold technique, conjoint analysis, and BWS (45).

Most patient-preference studies in healthcare research use stated-preference methods. Revealed-preference methods are only possible for existing products in the market, and thus are not applicable to novel medicines that are not yet available. Due to the hypothetically nature of stated-preferences, it has to be assumed that patients would actually choose the options they say they would. While revealed-preferences can avoid hypothetical bias, they still are subject to other bias, such as individual financial considerations. Furthermore, revealed-preference methods often are unable to infer the relative weights of individual attributes (37, 47, 48).

## Patient experience data use in medicines’ life cycle

### Patient reported outcomes

#### Medicine development and regulatory approval

PRO data obtained from clinical trials can inform the medicine development phase (e.g., study design), market authorization and post-authorization monitoring process, supporting the determination of benefit–risk balance and labeling claims in different moments of assessment (49). The patient perspective can complement traditional endpoints, such as objective clinical outcomes and laboratory parameters, which may not always fully capture the impact of a treatment. For instance, PRO data may help in disclosing treatment-related symptoms that need to be addressed or support choice between two medicines with a similar efficacy profile (50). As an example, overall survival (OS) is a common outcome assessed in randomized controlled trials (RCTs) to measure the clinical benefit of an experimental intervention, especially in oncology. However, this evaluation overlooks the experience of the patient during treatment (51). Consequently, these studies often include in the analysis the use of a PRO to assess health-related quality of life (HRQoL). Thus, a more holistic approach of the treatment might be evaluated, complementing other physiologic or clinical endpoints (48). PRO data can also be integrated into medical product labeling to provide information on safety and tolerability, and to support specific claims of treatment benefits. This can be relevant considering that the patients’ have first-hand knowledge of the effects of a certain medicine (12, 13, 52).

Several initiatives have been developed to promote best methodological practice for use of PRO in clinical trials. The PROTEUS Consortium, a project funded by the pharmaceutical industry, has identified core documents on designing PRO protocols (53), selecting PROM (50), analyzing PRO data (54), reporting PRO findings (55), among other aspects. These tools have been aggregated and described in detail in the PROTEUS Handbook (56). Selected PRO related guidance documents and tools are briefly described in the table below (Table 4).

**Table 4:** Selected documents and tools for PRO use in medicine development and approval.

Documents/tools	Description
CONSORT-PRO Extension (Consolidated Standards of Reporting Trials Statement-PRO extension) (44)	The CONSORT-PRO Extension was published in 2013 aiming to improve reporting of RCT PRO findings. It includes a five-item checklist on what should be included when reporting RCTs in which PROs are primary or secondary endpoints.
International Society for Quality-of-Life Research (ISOQOL) Guidance (42, 48)	In 2013, ISOQOL published a guidance on minimum standards for PROM in patient-centered outcomes and comparative effectiveness research. These standards can inform the selection of PROM for the respective research studies. In the same year, ISOQOL also developed a set of recommended standards for reporting RCT PRO results.
SPIRIT-PRO Extension (Standard Protocol Items: Recommendations for Interventional Trials in Patient-Reported Outcomes) (41, 46)	Released in 2018, the SPIRIT-PRO Extension comprises a 16-item checklist detailing the specific PRO content that should be incorporated into protocols. Its objective is to enhance the comprehensiveness and transparency of clinical trial protocols, particularly those where PROs are primary or key secondary endpoints. The authors advocate for utilizing the SPIRIT-PRO Extension in conjunction with the SPIRIT 2013 Guidelines, which establish the minimum content requirements for a clinical trial protocol.
SISAQOL (Setting International Standards in Analyzing Patient-Reported Outcomes and Quality of Life Endpoints Data) Consortium (43)	In 2021, the SISAQOL Consortium released initial recommendations for standardizing the analysis and interpretation of PRO and quality of life data in cancer clinical trials. These recommendations intend to facilitate the development of international consensual standards for PRO analysis in cancer RCTs.
Cochrane Handbook for Systematic Reviews of Interventions—Chapter 18 “Patient-reported Outcomes” (27)	The version 6.4 of this handbook, published in 2023, pretends to give authors tools to approach PRO in systematic reviews, by explaining how PROM are developed and by highlighting the importance of indicating unequivocally the PROM to measure the outcomes of interest.
Current Practices and Challenges When Submitting Patient Experience Data for Regulatory Decisions by the US Food and Drug Administration: An Industry Survey (57)	Survey aimed at assessing practices and challenges regarding PED, and PRO, in particular, submission for FDA regulatory approval.

In recent years, regulators have been paying more attention to PROs, especially in their medicine’s approval processes due to the rising interest of this area of knowledge. A review of 497 European Public Assessment Reports (EPAR) of authorized medicines and 19 EPARs of withdrawn medicines, published from 2017 to 2022, found that 48.3% and 52.6% stated use of PRO, respectively. In this study, PROs were mostly considered as secondary (53.3%) and exploratory endpoints (18.8%); in 32.5% of the cases, the PROs used were related to general quality of life; and PRO use was particularly low in some therapeutic areas, such as infectious diseases (15.2%) (58).

Considering an example from the oncology field, the results from a study conducted to evaluate the use of PROs for the approval of oncology medicines in the EU, from 2017 to 2020, demonstrated that out of the 104 clinical trials conducted for the approval of the 76 medicines studied, PROs were considered as a secondary endpoint in 57.7% and exploratory in 29.8%. These 76 medicines corresponded to 128 indications, however only in 22 of them the PROM use was mentioned in section 5.1 of the Summary of Product Characteristics (SmPC). This emphasizes how the contribution of PROs to evidence accepted for establishing the positive benefit–risk assessment of a new medicine can be challenging, mainly due to aspects related to study design, PROM selection and missing data (59).

Finally, a study looking into establishing the use of PROs in the approval of orphan medicines, revealed that, in the European context, PRO use in orphan medicines was lower when compared with all medicines’ approval. Nonetheless, when compared to the results from a similarly conducted study in the United States of America, the authors state that FDA approvals included significantly less PROMs in their processes in comparison with the EMA approvals, during the study period (60). This could be due to a relatively longer experience within the European context following the publication in 2005 of the HRQoL guidance, which suggests that regulatory guidance can stimulate the use of PROs (60, 61).

#### Health technology assessment

It is also necessary to progress toward a more patient-centric evaluation of health technologies (62, 63). In other words, some authors argue that there should be a reframing of the concept of health value that includes what patients perceive as value (63). In the HTA setting, the perception of value differs from the need of positive results of critical endpoints assessed by regulators. Particularly, HTA focus on comparative analysis to guarantee that the intervention reimbursed has the ability to diminish burden in the system, while facilitating access. In this sense, there is an opportunity to use PROMs in HTA to gather information on a treatment’s added value, which, in turn, can inform market access, reimbursements, and pricing negotiations. Incorporating PRO data in HTA can be essential in assessing the effectiveness and value of health technologies, and ultimately improve efficiency in resource allocation (28, 62).

A study analyzed HTA appraisal reports that contained PROs as endpoints between January 2018 and March 2020 and that were submitted to the Canadian, French, German, Scottish and British HTA agencies. PRO data were found in 77% (48 out of 62) of the reimbursement submissions (62 medicinal products assessed in total). PROs were included in 23 appraisals as a primary or a key secondary endpoint and 43% of these assessments (10 out of 23) received approval for reimbursement from at least three agencies. However, the authors report that most of the PRO data submitted received unfavorable assessments from various agencies. The main reasons listed were the absence of a predefined analysis for responders, utilization of a non-validated tool for collecting PROs, uncertainty in PRO measurements and meaningful changes in scores. Thus, the study showed that there is a considerable variability in HTA assessment of PROs and there is room for companies to better prepare their submissions (29).

#### Real-world setting

Conventionally, PRO data are more often collected in clinical trials to support regulatory, HTA and clinical decision-making. However, stakeholders are broadening their interest in better understanding the patient perspective in the real-world (routine clinical care) setting where PRO data collection has been of added value (17, 64). Considering a clinical care example, a study on patients who suffered acute stroke discharged from a tertiary care hospital demonstrated that the results of a clinician-reported outcome did not align with the patients’ perception obtained through PRO 3 months after the event (65).

Furthermore, real-world PRO data can also inform the early stages of medicine development. By using PROs to gather insights on the natural history of disease, disease burden and unmet needs, researchers can select the most suitable endpoints for the subsequent clinical trials. Finally, PROs may be a source of evidence in early access, compassionate use, and off-label use contexts (66).

A review of all publicly available data on post-authorization safety studies (PASS) protocols submitted to the Pharmacovigilance Risk Assessment Committee (PRAC), EMA and European Network of Pharmacovigilance and Pharmacoepidemiology (ENCePP) repositories, from 2012 to 2015, demonstrated that PRO use among regulatory medicines post-authorization safety assessment is still low. Almost half of the electronic register of PASS (EU-PAS) entries had the protocols available, of which only 14% included PRO data. Such PROs assessed disease burden, symptoms and quality of life (64).

In 2023, a study using qualitative interviews to assess the degree of PRO implementation in the real-world setting found consensus that the use of PRO in routine clinical care setting is not yet well established. The main challenges identified were lack of infrastructure and resources to collect PRO systematically in the real-world setting and few financing opportunities (67).

### Patient preferences

#### Medicine development and regulatory approval

Patient preferences (PP) information can address the relative weights between benefits and risks and what would be the maximum acceptable risk for a given health benefit, with such information being particularly useful in more complex benefit–risk evaluations for early access. Some reports suggest that information generated may be included on the product label to inform the patients regarding benefits and risks (39, 41, 68). The current regulatory thinking is that PP information can help healthcare professionals to identify situations of high preference heterogeneity and where an in-depth understanding of individual preferences is needed.

Despite the recognition of PP’s potential in regulatory decision-making, they are not yet systematically integrated in medicines’ life cycle, particularly at the medicine approval stage (39). In the literature, around 30 methods for incorporating PP are described, and some authors suggest that this number causes uncertainty for stakeholders when choosing a method and thus limiting its usage (69, 70). Additionally, in the 2022 workshop organized by the EMA, stakeholders recognized that PP studies might not have been used much in decision-making because they are complex and time consuming, and there is a lack of methodological harmonization. Stakeholders also encourage the development of PP tools based on practical experience to avoid rigidity in the implementation process of these methodologies and also of more guidance that might diminish this implementation’s hurdle (4, 70).

In 2015, the Innovative Medicines Initiative (IMI) launched the Patient Preferences in Benefit–Risk Assessments during the Drug Life Cycle project (Project PREFER), a collaborative effort between public and private entities such as the industry, academia, patient organizations, and a HTA organization. PREFER aimed to explore when and how PP should be considered to enhance decision-making process by regulatory and HTA organizations (71). This 6-year project was divided into work packages. The methodology work package was responsible for investigating the concerns stakeholders may have about using PP studies and provided recommendations on which methodologies should be used. The case study work package has conducted several studies based on the recommendations previously released. Finally, the recommendations work package launched a set of experience-based recommendations based on their work for PP inclusion throughout the medicines’ life cycle. Consequently, these recommendations are anticipated to aid in the formulation of guidelines for industry, regulatory and HTA institutions. From this project arose several publications, training materials, webinars to increase stakeholders’ familiarity with PP studies, and operational guidance and additional resources to assist in the design and implementation of these studies (71).

#### Health technology assessment

PP could inform HTA on non-health attributes that are not captured by traditional assessment tools (34, 39, 68, 72). In particular, PP can be useful in cost-effectiveness analysis balancing clinical outcomes and Quality-Adjusted Life Years (QALYs) and possibly in reducing uncertainty when it comes to adherence in the real-world setting (39).

Several HTA bodies, such as NICE in the UK, the German HTA body IQWiG, and the Belgian HTA body KCE, have shown growing interest in using patient preferences for HTA, and have engaged in projects to advance the field of PP assessment for HTA (40). However, despite this recognition, integration of PP in HTA processes remains a challenge and has not yet been widely used (72). Representativeness in the HTA process seems to be one of the limiting factors that hinder greater application of PP data, so more studies should be carried out in this regard (39).

#### Real-world setting

Throughout the post-marketing phase, PP may inform patient acceptability of a given therapy in routine clinical care settings, extensions of indications, post-marketing surveillance, specific treatment opportunities, need for optimizing existing medical products, and product innovation (39, 68, 73). For instance, as stated in the PREFER recommendations, a preference study could be set up to understand the patient acceptance of rare but serious safety signals observed in the post-approval phase. Finally, investigating revealed preferences in the real-world setting could add to the information provided by stated preferences and potentially improve external validity (43). Nonetheless, there are not yet many examples of the real application of PPs in the real-world setting, according to the literature. This limited incorporation of PP might be due to PP’s methodological challenges related to capturing and using real-world data for decision-making (limited access to databases and registries, data quality, complex analytical methodologies and confounding, for example) (74).

## Challenges and opportunities

### Regulatory guidance

As stated so far, the potential and number of applications of PED are vast. Moreover, all stakeholders seem to be aware of the importance of PED as a vehicle to develop a more patient-centric approach in healthcare and in the medicines’ regulatory field, in particular. This fact might be demonstrated by the number of past and ongoing efforts to leverage PE and PED in regulatory decision-making. In a review in 2023, 53 relevant global regulatory and HTA initiatives regarding the use of PED were highlighted (2). Additionally, this review showed that the majority of these initiatives (13) were of an international nature, followed by a tie between North America and Europe, which contributed equally with 11 initiatives (2).

In the European setting, EMA is demonstrating how this patient-centric vision has been put into practice by engaging patients and their representatives in its scientific committees’ and also in its public hearings (6). The “*Multi-stakeholder workshop: Patient experience data in medicines development and regulatory decision-making*” is also a recent evidence of the continuation of this vision and has provided essential multi-stakeholder reflections on how to progress integrating PED in the EU and addressing challenges in implementation (4, 75). In this context, it is also worth noting the possibilities of increasing the adoption of PED offered by the development of the European Health Data Space (76) and the reform of pharmaceutical legislation, both activities in progress at the time of writing this manuscript (77, 78).

In the US, FDA has developed an initiative called “Patient-Focused Drug Development” (PFDD) under the 21st Century Cures Act and The Food and Drug Administration Reauthorization Act of 2017 Title I. The PFDD initiative aimed at developing four guidances about methodological considerations that should be contemplated when submitting PED to FDA. Prior to these guidances, public workshops were organized and as Supplementary material, FDA published two other documents “*Submitting Clinical Trial Datasets and Documentation for Clinical Outcome Assessments Using Item Response Theory*” and “*Submitting Patient-Reported Outcome Data in Cancer Clinical Trials*” (79). Other stakeholders have also been able to share some feedback on these initiatives. In particular, various organizations (pharmaceutical industry, patient organizations, public institutions, professional associations) have provided comments on the methodological challenges related to PED as requested by the FDA (80). In addition, the Biotechnology Innovation Organization (BIO) also issued a white paper with recommendations for the PED table included in FDA review documents (81).

It is clear that regulatory agencies, namely EMA and FDA are engaged in efforts to create a more prepared ecosystem for the challenges posed. The main initiatives of interest that intend to promote and facilitate the use of different types of PED, developed by the two agencies, both in Europe and in the US, are briefly presented in Supplementary Table 1.

In the HTA landscape, the implementation of the Joint Clinical Assessment in the European setting presents an opportunity for PE and, consequently, for the use of PED. The guidance on Patient and Healthcare Professional Involvement advocates for the inclusion of individual patients with collective disease expertise as external experts, thereby allowing them to provide insights about treatment pathways, evaluate study designs and support the definition of the PICO framework for assessments. According to the same guidance, data derived from this involvement is expected to be collected through questionnaires, interviews and consolidated meetings (8). Despite the limited large-scale experience with these guidelines, there will be no need for an extended wait for feedback, as the Joint Clinical Assessment becomes mandatory for oncology medicines and advanced therapy medicinal products (ATMPs) in the beginning of 2025 (82).

Despite these efforts, stakeholders are calling for more regulatory guidance to be developed. On one hand, the medicines developers are asking for harmonization of the concepts, definition of standard methods and investing in collecting these data, and for the data to have a concrete role in decision-making processes. On the other hand, patient advocates and patient organization representatives and other stakeholders are demanding more transparency on how PED is evaluated and whether/why it is accepted as evidence to establish the benefit/risk so that they can assess the impact of their involvement in shaping development plans and regulatory decision-making (1, 4, 35, 83).

### Methodological aspects

Methodological aspects still represent a considerable challenge for most stakeholders, which is one of the reasons why more guidance is requested. Missing data is a well-known problem in PED, particularly in PRO analysis, and it is defined by the SISAQOL-IMI Consortium, a collaborative project supported by both public and private organizations under the Innovative Health Initiative (IHI), as any “*data that would be meaningful for the analysis of a given research objective or estimate but were not collected*” (54). Therefore, the study protocol should describe how missing data will be accounted for in the analysis. The proportion and reasons for missing values should also be reported. For example, if only patients who feel better can complete the questionnaire, it will likely introduce a selection bias, resulting in misleading results (54, 84). The high rate of missing data has been attributed to the logistical difficulty and time-consuming implementation of PRO assessment in clinical trials, which is why it is often deprioritized (85). To overcome the barriers associated with missing data and improve completion rates, the scientific literature recommends several strategies, including: assuring that patients understand the purpose of the assessment and value the utility of PRO data; giving clear instructions to participants, and ensuring that physicians are also aware of the value of PROs and receive tutoring on the compilation and interpretation of data originated by PRO; assigning a person responsible for PRO data collection in a given study, checking for completeness of questionnaires, and ascertaining the reasons for missing values (13, 79, 86). The SISAQOL Consortium has issued a set of recommendations on how to manage missing data (54).

Regarding PP, researchers are developing efforts to establish which PP methods are more suitable for each stage in the medicine life cycle. From the 33 PP exploration and elicitation methods reviewed, a sample of 13 propitious reference exploration and elicitation methods were selected, which corresponded to the ones that were more likely to meet decision-makers’ criteria according to the study’s inquired experts. This thoughtful approach could assist both researchers and decision-makers in selecting PP instruments appropriate for the intended purpose, thereby reducing the initial burden associated with the decision-making process (70). This study is a good illustration of how stimulating the academic community can aid regulatory guidance to leverage the use of PED.

Participant burden for the collection of PED is another complex issue associated with the methodological inherent aspects, since it depends not only on the frequency and timing of assessment, but also on the trial duration, length and/or formatting of the instrument, mode of administration (paper, telephone or web-based), literacy level, the complexity of instructions, and disease severity and/or treatment toxicity (87, 88). An excessive respondent burden may result in unwillingness to complete the questionnaires and, ultimately, will result in missing data. Therefore, extensive, and time-consuming surveys are generally unpractical (88, 89).

However, patient education might be a tool to mitigate the challenge previously mentioned, since educating individual patients or even carers may enhance their understanding of the importance of filling in a questionnaire regardless of its size. Moreover, this practice can minimize the risk of information bias. In turn, the challenge is to provide sufficient information on the patients’ role and the purpose of their input without being too burdensome, and without creating selection bias (68). Engaging with patients involved in representative associations, who are generally better informed due to training programs provided by patient organizations they are part of, may be an enticing solution to overcome the question of whether patients are properly educated.

It is worth highlighting an initiative by the International Council on Harmonization (ICH) that could represent a great opportunity, at a global level, to develop tools both capable of harmonizing PED concepts and understandings, and of responding to the methodological limitations raised by researchers and stakeholders. Concretely, in 2021, the ICH issued a reflection paper entitled “Proposed ICH Guideline Work to Advance Patient Focused Drug Development,” anticipating new ICH guidelines (90). In addition to identifying critical areas where integrating the patient’s perspective could enhance drug development and inform regulatory decision-making, this reflection paper outlines specific strategies for the development of two new ICH guidelines: (1) a guideline on “*what to measure in a clinical trial, including refining the set (list) of important impacts and concepts from patients, to select, modify or develop clinical outcome assessments (COAs) that can demonstrate change and define endpoints and meaningful change*”; (2) a guideline on “*methods for elicitation/collection, analysis, reporting and application of qualitative or quantitative assessments of the relative desirability or acceptability to patients of specified alternatives or choices among outcomes or other attributes that differ among the alternatives*” (90). When implemented, it is expected that the guidelines will have the capacity of fostering global harmonization of PED, so these can be collected, analyzed and used in a way that is methodologically robust and suited for patient groups collecting PED, the pharmaceutical industry, regulatory agencies and even healthcare systems overall (90).

### Digitalization

Technology and digitalization provide a unique opportunity for boosting passive and active patient data collection. For instance, some applications provided by personal devices (smartphones and smartwatches) can passively collect relevant data to inform a trial. These devices can unlock even greater potential when combined with medical devices or active data collection methods to fully understand patients’ experiences with a disease and/or treatment. However, ethical constraints, such as collection of sensitive data and GDPR, may present challenges that need to be overcome (91).

The possibilities offered by mobile health (mHealth) have increased over the last few years with the expanding use of phones and wearables. Consequently, regulatory agencies and medicine developers have been considering them for several phases of product development, such as clinical trials and pharmacovigilance activities. Since technological devices have lots of versatility, they have the benefit of being adaptable to a wide range of concepts of interest. Moreover, they can collect tremendous amounts of data in real time, when compared with traditional data collection measures (92), while reducing the burden of data collection on the patient. Although, PED coming from these advanced technologies could be of relevance in clinical trials setting, understanding how these data can be validated, used and interpreted is paramount when submitting applications/scientific data for regulatory assessment (66). In post authorization environments, mHealth can be used for pharmacovigilance purposes. For example, IMI WEB-RADR launched a mobile phone app that enables healthcare professionals and patients to report spontaneous AE reports directly to the competent authorities. Another example is the Med Watcher app, supported by the FDA, which has been used to improve the efficiency of reporting individual case safety reports (93).

Digitization also includes using social media as a potential data source for PED. Social media provide an easy platform for people and patients to exchange information, experiences, and opinions, along with the rise of new possibilities for patients to get more information on their diseases and treatments, and has contributed to the increase of new online patient groups who discuss health-related matters and that can also interact with healthcare providers and even academics (94). An example of social media contributing patient experience to define burden of a new disease in a crisis setting is Long COVID, where the patient experience reported in Twitter helped to start patient advocacy and scientific discussion on this new condition (95). Social media may hold potentially valuable safety information in several contexts (96–98), detecting undisclosed signals, capturing less frequently reported AE, or detect AE earlier than traditional methods. In addition to signal detection, social media could also detect the potential for abuse or misuse of certain medications, evaluate acceptability and risk perception by patients, as well as provide a validation tool for signals reported in other surveillance systems (99–102).

Yet, the validity of information posted online by patients requires careful analysis and interpretation, as patients may misinterpret and/or misreport their diagnosis, clinical outcomes, symptoms, or treatment regime (98, 103, 104). Given the volume and complexity of this data, the development and standardization of more effective and robust mining and processing methods is necessary and is yet to be established. The enhancement of mining strategies may provide higher quality data, potentially broaden the scope and utility of social media, provide more meaningful results, and reduce the burden of storage and analysis (100–102). Moreover, the population using social networks cannot be seen as representative of the overall population, since the demographics of individuals using these channels are barely known and may differ across different social media networks (96, 105). Although in recent years, users aged over 75 are getting more engaged with social media, users tend to be younger adults, with higher education and less ill or functionally disabled (96, 105).

In conclusion, digitalization has boosted the development of mHealth, and the processing of data extracted from social media. To fully use this data in regulatory decision-making, compliance with ethical and legal considerations need to be ensured; a sound regulatory framework should be in place and any methodological challenges that arise need to be overcome through further research (93). Despite all the additional challenges, the use of these data offers an opportunity to engage patients in a completely new way, providing insights that may have never been obtained before with conventional data collection methods.

## Conclusion

This review tries to present an overview of the status of PED and potential for use in different settings of medicines’ life cycle at the time of this publication, including discussing challenges and opportunities to maximize regulatory use of PED. All parties acknowledge the importance of ensuring that patients’ views, values and preferences about the effects of a medicine are an essential part of the information at the early stages of the development and in any subsequent decision about its authorization and use.

Regulatory agencies and other stakeholders recognize PED integration into decision-making as a strategic priority, acknowledging the paramount importance of placing the patients’ preferred outcomes and their perspectives at the forefront of medicines development and evaluation. Increasing patient engagement initiatives have not only encouraged discussions on the use of PED, but also hold the potential to promote the generation and collection of such valuable data. Furthermore, these patient engagement initiatives could contribute to patient advocacy strategies, which in turn could enhance the medicines regulatory field by providing new and thoughtful insights. This creates a self-sustained cycle of sharing, promoting and developing good practices regarding the use of PED among the different stakeholders involved, including patient organizations. These organizations play a crucial role in liaising these initiatives and serving as a focal contact point between all interested parties.

Still, there is a long way to go to reach optimal use of PED in every stage of a medicine’s life cycle. This literature review indicates that PROs are the type of PED where experience to date is more established and are more advanced in implementation, quite possibly due to their intrinsically more quantitative nature. In addition, current evidence indicated that there is more research and regulatory guidance on PROs than for other types of PED, especially in the medicine development and regulatory approval settings. Nevertheless, PRO data are no substitute for PP data, which bring a different contribution to the scene by allowing patients, based on their own benefit–risk analysis, to opt for a particular choice. Even so, the evidence seems to suggest that PP data needs further research to achieve greater potential for its use in regulatory processes.

Several initiatives are ongoing at a global level, implemented by the various stakeholders, particularly by regulatory agencies, to enhance the use of PED in decision-making, while taking into consideration how PED can also contribute to HTA and clinical decisions. However, stakeholders are calling for additional efforts as there are challenges that have not yet been overcome. It is important to harmonize both the materials already published and the knowledge extracted from them, while also presenting solutions to the methodological challenges inherent to these data’s nature. In this context, the publication of the ICH guidelines could mark the first step along this path. On the other hand, digitalization has put the discussion of generating PED through mHealth and social media on the table. Although these bring their own challenges, the opportunity offered by these methodologies cannot be neglected in order to generate data that otherwise, i.e., conventionally, would not be obtained. The academic community could also play a crucial role in solidifying the landscape around PED, given the transversal added value it provides, especially in terms of addressing methodology-based obstacles. Hence, further research on the development and validation of adequate methods should be encouraged. Ongoing work in the EU on a reflection paper in PED is expected to encourage stakeholders to collect and submit PED to medicines regulators, offering the existing pathways of scientific advice and qualification of novel methodologies as the best platform to discuss early a company’s study design development plans using PED.

Although still under consultation, the new EU’s pharmaceutical legislation proposal (106) emphasizes the need for increased participation by patients in decision-making and treatment optimization. Once adopted, it will be important to review how the new provisions will impact the evolution of the patient experience data field and how PED will fit into any future regulatory processes and outcomes. What is beyond doubt is the unanimous agreement of all stakeholders that high quality data reflecting the direct experience from patients can be meaningful for regulatory purposes and in healthcare decisions if robust methodologies are used to collect and analyze these data. To get there, we should leverage the current momentum to solidify the PED framework in order to guarantee its success in the realm of regulatory decision-making. With all the initiatives outlined and planned, and by fostering a collaborative and constructive spirit among stakeholders, challenges will turn into opportunities, leveraging PED use in regulatory decision-making.

## Author contributions

DA: Formal analysis, Investigation, Writing – original draft. DU: Project administration, Validation, Writing – review & editing. RG-Q: Project administration, Validation, Writing – review & editing. AA: Investigation, Writing – review & editing. JB: Project administration, Supervision, Validation, Writing – review & editing. PV: Supervision, Validation, Writing – review & editing. NB: Conceptualization, Writing – review & editing. BS: Conceptualization, Supervision, Validation, Writing – review & editing. CT: Conceptualization, Methodology, Project administration, Supervision, Validation, Writing – review & editing.
