# Supplementary material for: Leveraging patient experience data to guide medicines development, regulation, access decisions and clinical care in the EU
Source: Front Med (Lausanne). 2024 May 23;11:1408636. doi: 10.3389/fmed.2024.1408636 (PMC11153762; doi:10.3389/fmed.2024.1408636)
Supplement: Supplementary file 1 [file Table_1.docx]

# Supplementary material

**Table 1.** Selected guidance and other initiatives regarding PED use in regulatory decision-making.

| **EMA** | |
| --- | --- |
| **Documents** | **Objective** |
| **Reflection Paper on the Regulatory Guidance for the Use of Health-Related Quality of Life (HRQL) Measures in the Evaluation of Medicinal Products** (July 2005) (1) | To discuss the use of HRQoL in the medicine development process. |
| **EMA Benefit-Risk Methodology Project** (March 2009) (2) | To identify decision-making models applicable to the Agency's processes, aimed at enhancing consistency, transparency, and auditability in assessing the benefits and risks of medicines. Patient experience data are referred as key measures to be considered in the benefit-risk assessment. |
| **Qualification of Novel Methodologies for Medicine** **Development: Guidance to Applicants** (January 2009, updated 2014) (3) | To describe the process for sponsors and researchers to obtain medicine development tools (DDT) qualification expeditiously, which is also applicable for novel PROM. |
| **Appendix 2 to the Guideline on the Evaluation of Anticancer Medicinal Products in Man: The Use of Patient-reported Outcome (PRO) Measures in Oncology Studies** (April 2016) (4) | To reflect on the principles of scientific best practice regarding PRO validation, selection, or application in oncology studies from a regulatory perspective. |
| **EMA’s Regulatory Science to 2025 strategy** (March 2020) (5) | This strategy has recommended incorporating patient preferences to guide benefit-risk assessment and acknowledges the need to develop guidance based on recent studies and initiatives to inform the implementation, analysis and identification of potential roles of preference studies, with the ultimate goal to enable a systematic and structured integration of patient preferences in medicines’ life cycle. |
| **Multi-stakeholder workshop: Patient experience data in medicines development and regulatory decision-making** (September 2022) (6) | To establish a common understanding of patient experience data, assess current methods of data collection and incorporation into medicine development and regulation, explore leveraging direct patient data from real-world healthcare, and agree on priorities for enhancing the collection and use of patient experience data.  The recorded workshop is available through the following link: https://www.ema.europa.eu/en/events/multi-stakeholder-workshop-patient-experience-data-medicines-development-and-regulatory-decision-making |
| **European medicines agencies network strategy to 2025 – Protecting global health at a time of rapid change** (December 2020) (7) | To define priority areas and strategic goals to guide the EMA’s action. Patient experience data is addressed in the document as a priority to achieve and improve patient-centred decision-making both in medicine development and regulation. |
| **European medicines agencies network strategy to 2025: Mid-point report to Q2 2023** (December 2023) (8) | To provide the key achievements obtained so far with the implementation of the strategy. Regarding patient experience data, the report highlights:   - Organisation of a multi-stakeholder workshop on patient experience data. - Establishment of an internal working group on patient experience data. - Call for expressions of interest for EU Network experts to join an expert group to work on a reflection paper about the topic. - Drafting of the reflection paper. - Development of an Action Plan on patient experience data. |
| **FDA** | |
| **Documents** | **Objective** |
| **Patient-Reported Outcome Measures: Use in Medical Product Development to Support Labelling Claims. Guidance for Industry** (December 2009) (9) | To provide considerations on PRO instrument development and trial design for labelling claims. |
| **Roadmap to Patient-Focused Outcome Measurement in Clinical Trials** (August 2015) (10) | To help researchers develop new or adapt existing tools, including PROM, that measure outcomes that matter most to patients |
| **Qualification Process for Drug Development Tools** (October 2020) (11) | To describe the process for sponsors and researchers to obtain qualification of novel drug development tools (DDT)s from the Agency, including PROM. |
| **Patient-Focused Drug Development (PFDD) Initiative** (2020 – present) (12) | To address how stakeholders can gather and submit comprehensive and pertinent patient experience data for drug development and evaluation. Compilation of four guidelines:   - Guidance 1: Collecting Comprehensive and Representative Input. - Guidance 2: Methods to Identify What is Important to Patients. - Guidance 3: Selecting, Developing or Modifying Fit-for-Purpose Clinical Outcomes Assessments. - Guidance 4: Incorporating Clinical Outcome Assessments into Endpoints for Regulatory Decision Making. |
| **Core Patient-Reported Outcomes in Cancer Clinical Trials: Draft Guidance for Industry** (June 2021) (13) | To provide additional considerations on use of PROM in cancer clinical trial setting. |
| **Submitting Patient-Reported Outcome Data in Cancer Clinical Trials** (November 2023) (14) | To provide technical specifications for submitting PRO data collected in cancer clinical trials to support a marketing application for a medical product in oncology. |
| **FDA: Patient Preference Information – Voluntary Submission, Review in PMAs, HDE Applications, and *de novo* Requests and Inclusion in Decision Summaries and Device Labelling: Final Guidance** (15) | Guidance issued to encourage the submission of patient preferences information to support FDA’s decision-making process, focused on medical devices (labelling). |
| **A Framework for Incorporating Patient Preferences Regarding Benefits and Risks into Regulatory Assessment of Medical Technologies** (16) | A framework assessing patient preferences and their potential use and value in the regulatory context and beyond, focused on medical devices (assessment). |

# References

1. European Medicines Agency. Reflection Paper on the Regulatory Guidance for the Use of Health- Related Quality of Life (HRQL) Measures in the Evaluation of Medicinal Products. (2005). https://www.ema.europa.eu/en/documents/scientific-guideline/reflection-paper-regulatory-guidance-use-health-related-quality-life-hrql-measures-evaluation-medicinal-products_en.pdf [Accessed January 25, 2024]

2. European Medicines Agency. Benefit-Risk Methodology Project - Development and testing of tools and processes for balancing multiple benefits and risks as an aid to informed regulatory decisions about medicinal products. (2009). https://www.ema.europa.eu/en/about-us/what-we-do/regulatory-science-research/benefit-risk-methodology [Accessed February 16, 2024]

3. European Medicines Agency. Qualification of novel methodologies for drug development: guidance to applicants. https://www.ema.europa.eu/en/documents/regulatory-procedural-guideline/qualification-novel-methodologies-drug-development-guidance-applicants_en.pdf [Accessed December 17, 2023]

4. European Medicines Agency. Appendix 2. Guideline on the evaluation of anticancer medicinal products in man The use of patient-reported outcome (PRO) measures in oncology studies. (2016). 1–18 p. https://www.ema.europa.eu/en/documents/other/appendix-2-guideline-evaluation-anticancer-medicinal-products-man_en.pdf [Accessed February 16, 2024]

5. European Medicines Agency. EMA Regulatory Science to 2025 Strategic reflection. (2020). https://www.ema.europa.eu/en/documents/regulatory-procedural-guideline/ema-regulatory-science-2025-strategic-reflection_en.pdf [Accessed December 17, 2024]

6. European Medicines Agency (EMA). Patient experience data in EU medicines development and regulatory decision-making: Outcome of the workshop on 21st September 2022. (2022). https://www.ema.europa.eu/en/documents/other/executive-summary-patient-experience-data-eu-medicines-development-and-regulatory-decision-making-workshop_en.pdf [Accessed October 27, 2023]

7. European Medicines Agency. European medicines agencies network strategy to 2025 - Protecting public health at a time of rapid change. (2020). https://www.ema.europa.eu/en/about-us/how-we-work/european-medicines-regulatory-network/european-medicines-agencies-network-strategy [Accessed February 16, 2024]

8. European Medicines Agency. European medicines agencies network strategy to 2025 - Mid-point report to Q2 2023. (2023). https://www.ema.europa.eu/en/about-us/how-we-work/european-medicines-regulatory-network/european-medicines-agencies-network-strategy [Accessed February 16, 2024]

9. Food and Drug Administration. Guidance for Industry Patient-Reported Outcome Measures: Use in Medical Product Development to Support Labeling Claims. (2009). https://www.fda.gov/media/77832/download [Accessed April 29, 2022]

10. Division of Clinical Outcome Assessment, U.S. Food and Drug Administration. Roadmap to Patient-Focused Outcome Measurement in Clinical Trials (text version) | FDA. (2018) https://www.fda.gov/drugs/drug-development-tool-ddt-qualification-programs/roadmap-patient-focused-outcome-measurement-clinical-trials-text-version [Accessed February 14, 2022]

11. U.S. Food and Drug Administration. Qualification Process for Drug Development Tools -Guidance for Industry and FDA Staff. (2020). https://www.fda.gov/media/133511/download (2020). [Accessed February 14, 2022]

12. Food and Drug Administration (FDA). FDA Patient-Focused Drug Development Guidance Series for Enhancing the Incorporation of the Patient’s Voice in Medical Product Development and Regulatory Decision Making. https://www.fda.gov/drugs/development-approval-process-drugs/fda-patient-focused-drug-development-guidance-series-enhancing-incorporation-patients-voice-medical [Accessed January 16, 2024]

13. U.S. Food and Drug Administration. Core Patient-Reported Outcomes in Cancer Clinical Trials Guidance for Industry – Draft Guidance. (2021). https://www.fda.gov/media/149994/download [Accessed February 14, 2022]

14. Food and Drug Administration. Submitting Patient-Reported Outcome Data in Cancer Clinical Trials: Guidance for Industry - Technical Specifications Document. (2023). https://www.fda.gov/regulatory-information/search-fda-guidance-documents/submitting-patient-reported-outcome-data-cancer-clinical-trials [Accessed December 11, 2024]

15. U.S. Food and Drug Administration. Patient Preference Information – Voluntary Submission, Review in PMAs, HDE Applications, and De Novo Requests and Inclusion in Decision Summaries and Device Labeling: Final Guidance. (2016) https://www.fda.gov/media/92593/download [Accessed February 17, 2024]

16. Ho M, Saha A, McCleary KK, Levitan B, Christopher S, Zandlo K, Braithwaite RS, Hauber AB. A Framework for Incorporating Patient Preferences Regarding Benefits and Risks into Regulatory Assessment of Medical Technologies. *Value in Health* (2016) 19:746–750. doi: 10.1016/j.jval.2016.02.019

## 
